# ASSOCIATIONS OF ACTIGRAPHIC SLEEP WITH ADIPOKINES IN OLDER ADULTS

**DOI:** 10.1093/geroni/igad104.1829

**Published:** 2023-12-21

**Authors:** Yiwei Yue, Zhikui Wei, Jill Rabinowitz, Chee Chia, Toshiko Tanaka, Eleanor Simonsick, Luigi Ferrucci, Adam Spira

**Affiliations:** Johns Hopkins Bloomberg School of Public Health, Baltimore, Maryland, United States; Johns Hopkins School of Medicine, Baltimore, Maryland, United States; Johns Hopkins Bloomberg School of Public Health, Baltimore, Maryland, United States; National Institute on Aging, National Institutes of Health, Baltimore, Maryland, United States; National Institute on Aging, National Institutes of Health, Baltimore, Maryland, United States; National Institute on Aging, National Institutes of Health, Baltimore, Maryland, United States; National Institute on Aging, National Institutes of Health, Baltimore, Maryland, United States; Johns Hopkins Bloomberg School of Public Health, Baltimore, Maryland, United States

## Abstract

Adipokines are adipose-derived cytokines that play important roles in metabolism and obesity. Accumulating evidence suggests that sleep disturbance could alter adipokine levels and contribute to systemic metabolic dysfunction in younger and middle-aged populations. Less is known about links between sleep disturbance and adipokines in older adults. We examined cross-sectional associations of actigraphic sleep parameters with adipokine levels in 353 community-dwelling older adults aged 74.8±8.3 years (51.3% male, 26.6% non-white) enrolled in the Baltimore Longitudinal Study of Aging. Participants completed 6.56±1.06 nights of wrist actigraphy and had morning fasting adipokine levels measured in plasma. Predictors included actigraphic total sleep time, sleep onset latency, wake after sleep onset, sleep efficiency, and average wake bout length (WBL), and outcomes were levels of the adipokines, specifically adiponectin and leptin. Adjusting for sex, age, race, years of education, BMI, hypertension, and diabetes, longer WBL was associated with lower adiponectin (B=-1.73, 95%CI: -3.07,-0.38) and leptin (B=-2.93, 95%CI: -4.61,-1.24). WBL interacted with sex (p-value for interaction = 0.018); higher WBL was associated with lower adiponectin in men (B=-4.12, 95%CI: -6.46, -1.79), but not in women (B=-0.34, 95%CI: -2.48,1.80). Findings link sleep fragmentation to lower leptin and adiponectin in older adults, particularly lower adiponectin in older men. Findings provide a potential mechanism by which disturbed sleep may promote metabolic derangements in later life. Prospective studies and sleep intervention trials are needed to evaluate whether these associations are causal, and to elucidate factors contributing to sex differences in these associations.

